# The Association between Elevated Levels of Peripheral Serotonin and Its Metabolite – 5-Hydroxyindoleacetic Acid and Bone Strength and Metabolism in Growing Rats with Mild Experimental Chronic Kidney Disease

**DOI:** 10.1371/journal.pone.0163526

**Published:** 2016-10-06

**Authors:** Dariusz Pawlak, Ewa Oksztulska-Kolanek, Beata Znorko, Tomasz Domaniewski, Joanna Rogalska, Alicja Roszczenko, Małgorzata Michalina Brzóska, Anna Pryczynicz, Andrzej Kemona, Krystyna Pawlak

**Affiliations:** 1 Department of Pharmacodynamics, Medical University of Bialystok, Bialystok, Poland; 2 Department of Monitored Pharmacotherapy, Medical University of Bialystok, Bialystok, Poland; 3 Department of Toxicology, Medical University of Bialystok, Bialystok, Poland; 4 Department of General Pathomorphology, Medical University of Bialystok, Bialystok, Poland; Universite de Lyon, FRANCE

## Abstract

Chronic kidney disease (CKD) is associated with disturbances in bone strength and metabolism. The alterations of the serotonergic system are also observed in CKD. We used the 5/6 nephrectomy model of CKD to assess the impact of peripheral serotonin and its metabolite– 5-hydroxyindoleacetic acid on bone biomechanical properties and metabolism in growing rats. The animals were sacrificed one and three months after nephrectomy. Biomechanical properties were determined on two different bone types: the cortical bone of the femoral diaphysis using three-point bending test and the mixed cortico-trabecular bone by the bending test of the femoral neck. Biomechanical tests revealed preserved cortical bone strength, whereas work to fracture (W) and yield load (F_y_) of mixed cortico-trabecular bone were significantly lower in CKD compared to controls. Serum activity of alkaline phosphatase (ALP), a bone formation marker, and tartrate-resistant acid phosphatase (TRACP 5b) reflecting bone resorption, were similar in CKD and controls. ALP was associated with lower femoral stiffness and strength, and higher displacements and W. TRACP 5b was inversely associated with cortical F_u_ and W. The elevated peripheral serotonergic system in CKD was: inversely associated with stiffness but positively related to the displacements and W; inversely associated with cortical F_y_ but positively correlated with this parameter in cortico-trabecular bone; inversely associated with ALP in controls but positively correlated with this biomarker in CKD animals. In conclusion, this study demonstrates the distinct effect of mild degree of CKD on bone strength in rapidly growing rats. The impaired renal function affects the peripheral serotonin metabolism, which in turn may influence the strength and metabolism of bones in these rats. This relationship seems to be beneficial on the biomechanical properties of the cortico-trabecular bone, whereas the cortical bone strength can be potentially reduced.

## Introduction

Serotonin (5-hydroxytryptamine, 5-HT) regulates a wide range of physiological processes: mood, perception, appetite, cognition, pain sensitivity, thermoregulation, sleep, sexual behavior, and circadian rhythm [1–6]. Serotonin is synthesized from the essential amino acid tryptophan (TRP) in the catalytic action of tryptophan hydroxylase (Tph), which in vertebrates has two isoforms, Tph-1 and Tph-2 [4–5]. Tph-1 catalyzes peripheral serotonin biosynthesis and is mainly expressed in non-neuronal tissues such as enterochromaffin cells of the gut that synthesize almost 90% of peripheral 5-HT [4]. Moreover, a very small amount of serotonin is also synthesized in bone tissue [5].

Recently, serotonin has received intensive attention due to its potential role in bone metabolism [7]. However, the issue of 5-HT and bone biology is still controversial, and is closely dependent on the site of its synthesis: 5-HT released from the duodenum inhibits osteoblast activity and decreases bone formation, while brain serotonin has an osteoanabolic effect [6, 8–9]. Yadav et al. [10–12] argue that peripheral 5-HT is a powerful inhibitor of osteoblast proliferation and bone formation without any effect on bone resorption. They also showed that pharmacological inhibition of Tph-1 was able to prevent bone loss in ovariectomized (OVX) animals [10]. Animal and human studies confirmed that higher levels of circulating serotonin may increase bone turnover and reduce bone formation [7–8, 10, 13–15]. The increased peripheral serotonin levels are observed during the development of osteoporosis in OVX animal model [15–16], which is widely used for investigation of postmenopausal osteoporosis. Moreover, patients and animals treated with selective serotonin reuptake inhibitors (SSRIs) or serotonin-norepinephrine reuptake inhibitors (SNRIs) have increased risk of bone fracture, based on high bone turnover markers and low bone mineral density [4, 17–22].

On the other hand, patients with carcinoid syndrome, who had elevated levels of circulating 5-HT and higher urinary excretion of its metabolite– 5-hydroxyindoleacetic acid (5-HIAA), showed no differences in bone density and microarchitecture, compared to healthy controls [23–24]. The long-term 5-HT subcutaneous administration led to higher bone mineral density, cortical thickness and femoral stiffness in rats compared to non-treated controls [25].

The changes in bone metabolism and microarchitecture are frequently observed in patients with chronic kidney diseases (CKD) [26–28] and in experimental models of chronic renal insufficiency [29–32]. Disturbances in mineral metabolism are common during CKD and have been classified as a new clinical entity known as CKD-Mineral and Bone Disorders (CKD-MBD) [33–34]. Interestingly, CKD-MBD syndrome may begin early in the course of kidney disease [35] and is characterized by secondary hyperparathyroidism, hyperphosphatemia, impaired bone metabolism, strength and increased risk of fracture [30–33]. Moreover, the previous investigations in our group [36–38] and by others [26, 39–46] revealed the alterations of the peripheral serotonergic system among patients and rats with CKD. These findings have become the basis for the hypothesis that disturbances in the peripheral serotonergic system may affect the bone metabolism and strength in a course of CKD.

Until now, there are few studies, in which bone strength were analyzed using biomechanical testing in the course of CKD [47–48]. Therefore, we performed subtotal nephrectomy—an experimental model, which mimics human CKD, to investigate the impact of serotonin and its metabolite– 5-HIAA on bone biomechanical properties as well as on bone metabolism in the course of CKD. Because the childhood and adolescence are critical periods for acquiring bone mass and strength [49], we used very young (one-month old) male Wistar rats during an intensive growth phase.

## Materials and Methods

### Animals

Forty-four male, 4 weeks old Wistar rats were purchased from and housed in the Center of Experimental Medicine of Medical University of Bialystok (Poland), according to Good Laboratory Practice rules. The animals were housed in conventional cages in vivarium conditions (temperature 22 ± 2°C °C, humidity 55 ± 5%, and a 12-h on/off cycle for lighting), and were allowed to have access to sterilized tap water and a standard rats’ chow (Ssniff R-Z V1324), containing 1% calcium, 0.7% phosphorus and 19% protein. The animals’ health status was monitored throughout the experiments by a health surveillance programme according to Federation of European Laboratory Animal Science Associations (FELASA) guidelines. The rats were free of all viral, bacterial, and parasitic pathogens listed in the FELASA recommendations. This study was carried out in strict accordance with ARRIVE guidelines, Directive 2010/63/EU of the European Parliament and of the Council on the protection of animals used for scientific purposes and the national laws. All the procedures involving animals and their care were approved by Local Ethical Committee on Animal Testing at the Medical University of Bialystok (Permit Number: 17/2012). All surgery was performed under sodium pentobarbital anesthesia (40 mg/kg, Morbital, Biowet Pulawy, Poland), and all efforts were made to minimize suffering.

### Experimental design, tissue collection and storage

After allowing a few days to adaptation to the new environment, the rats were randomly divided into two groups: the first, in which rats were subtotal nephrectomized (CKD, n = 22) and the second, in which the animals were sham-operated (CON, n = 22), at the age of one month. Surgery was performed by experienced operators according to the procedure described by Šviglerová et al. [50]. The subtotal nephrectomized rats underwent two-step surgical resection of 5/6 kidney, in a first-step a 2/3 of the functional left kidney was dissected and after 2 weeks the right kidney was completely removed. Shame operated rats (CON) experienced renal evacuation and decapsulation and then the intact kidney return into the abdominal cavity. That protocol was done twice, with two-week intervals. Blood samples and left femurs were collected one month after second surgery from 11 CKD and 11 control rats (CKD-1, CON-1), and 3 months after second surgery from 11 CKD and 11 control rats (CKD-3, CON-3), respectively. The number of animals in each experimental group was in agreement with the recommendation of Leppanen et al. [51]. It reduces the number of sacrificed animals according to the 3R rule, ARRIVE guidelines, Directive 2010/63/EU of the European Parliament and of the Council on the protection of animals used for scientific purposes and the national laws. At the end of each experiment, rats were placed in metabolic cages for 24-hours urine collection. The urine was aliquoted and frozen at -80°C until assays were performed. Next day, rats were weighted, anesthetized until unconscious and blood samples were taken from cardiac puncture. Rats were sacrificed by cutting the heart muscle. Taken blood was centrifuged for 10 minutes at 4000 x g to obtain serum. After centrifugation, serum was stored and frozen at -80°C until assays were performed. The kidneys and proximal tibiae were removed and fixed in 4% neutral formalin. Left femurs were dissected, cleaned of adhering soft tissue, and placed in the plastic tubes with physiological saline and frozen -20°C for biomechanical tests. This procedure does not affect bone’s biomechanical properties [52].

### Histological analysis

Formalin-fixed, paraffin-embedded kidney tissue was cut on a microtome to 4 μm thick sections and stained with hematoxylin and eosin. The analysis of microscopical findings was performed using semiquantitative method for the following histopathological indicators: the degree of renal glomerulosclerosis, tubular atrophy, interstitial fibrosis and interstitial lymphocytic infiltration [53].

The bone sections obtained from the proximal tibiae were decalcified in a solution of hydrochloric acid and formic acid according to Richman et al. [54]. The bone have been cut in the antero-posterior planes, and they have been fixed in 4% buffered formalin for 1 hour. Paraffin-embedded bones were cut to 4 μm thick sections and stained with hematoxylin and eosin. The osteoclasts number per mm^2^ was rated in ten 1x1 mm^2^ representative fields of view in the secondary spongiosa of the proximal tibia, starting between 1 and 2 mm distal to the growth plate, at a magnification of x200.

All histological evaluations of kidney and bone tissue were performed randomly in the light microscope (Olympus CX40) by two independent pathologists.

### Biomechanical testing

At the day of testing, the specimens were slowly thawed at room temperature and kept wrapped in the saline-soaked gauzes except during measurements. The biomechanical properties of left femurs were determined using three-point-bending test of femoral diaphysis and bending test of the femoral neck, as has been previously detailed [55].

**Fig 1:**
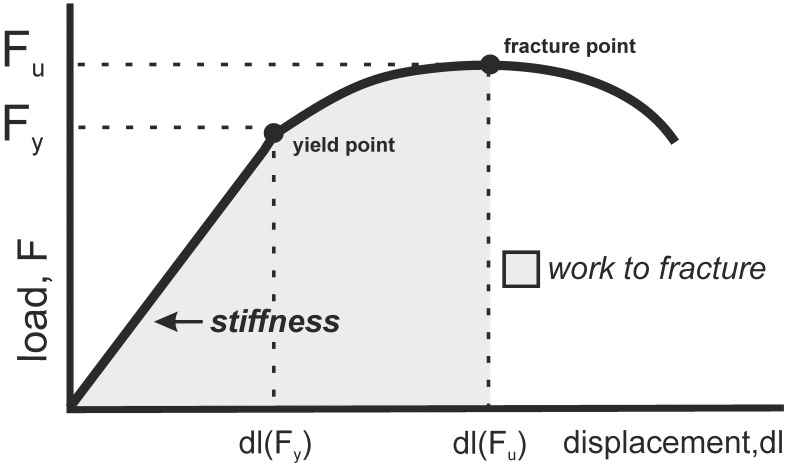
The schematic presentation of the biomechanical properties of an individual bone obtained in three-point bending test. Abbreviations: F_y_ = yield load, F_u_ = ultimate load, dl (F_y_) = displacement at the yield load, dl (F_u_) = displacement at the ultimate load, W = work to fracture.

Biomechanical testing was done with a testing machine Zwick Roell Z.2.5 (Germany) using testXpert II software. According to the producer, the measurement error of the method is ± 1% of the recorded value. All tests were performed on the same day by the same operator [55]. A load was applied to a bone midway between two supports separated by a constant distance of 18 mm. Bones were positioned so that loading point was at the center of femoral diaphysis, and bending occurred at the medial-lateral axis. The three-point bending test performed on femoral diaphysis reflects the biomechanical properties of the virtually pure cortical bone [48, 52]. Next, the proximal end of the femur (previously tested using the three-point bending test) was immobilized in an upright position in a hole of a stainless-steel block. The head of the femur was loaded vertically to the shaft until the femoral neck fractured [55]. The bending test of the femoral neck reflects the biomechanical properties of the mixed cortico-trabecular structure, in which the proportion of trabecular bone is about 25–30% [52]. The bone was loaded with a constant speed of 2 mm/minute in three-point-bending test and with 6 mm/minute in a bending test of the femoral neck. When increasing load acts on a bone, the bone first deforms linearly (reversible process) with the load up to so-called yield point (F_y_, yield load). Beyond the yield point, the bone deforms nonlinearly (irreversible process) until it fractures (F_u_, ultimate load). The load-displacement curves were recorded on-line and parameters analyzed were: F_y_, F_u_, the displacement at the yield load [dl (F_y_)] and the displacement at the ultimate load [dl (F_u_)], work to fracture (W) and stiffness (Fig 1). Each of these parameters reflect different properties of the bone: F_y_ reflects the force at the onset of first damage, representing the maximal whole-bone strength in elastic conditions, F_u_ indicates the minimum necessary load to fracture the bone in the assayed conditions and estimates the structural bone strength. W represents an amount of energy necessary to fracture the bone (bone toughness), and the displacements illustrate: dl (F_y_)–the maximal deformation of bone under elastic conditions (at yield point), and dl (F_u_)–the deformation at the fracture point. Stiffness was calculated from the linear portion of the load–displacement curve as a ratio of the load and displacement [55, 56–58].

### High Performance Liquid Chromatography

TRP and its metabolites (5-HT, 5-HIAA) were determined by high-performance liquid chromatography (HPLC). The chromatographic equipment was an Agilent Technologies 1260 series LC system composed of G1321 binary pump VL, G1379B degasser, G1329A autosampler, G1330B thermostat for autosampler, G1316A column thermostat, G1315C diode array detector, and Hewlett Packard HP1046A electrochemical detector.

Deproteinized samples were prepared by adding 20μl of 2M perchloric acid into the 100μl of serum. Urine samples were first diluted with distilled water (1 part of urine + 9 parts of water). The acidified samples were vortexed, kept at 4°C for 20 min, and then centrifuged for 30 min at 14000 x g at 4°C. Two μl of the supernatant was injected into HPLC system for analysis. The prepared sample was separated on ODS column (Waters Spherisorb 3μm ODS 2, 2.1x150 mm). The column effluent was monitored with diode array detector TRP—260nm. The mobile phase was composed of 0.1M acetic acid, 0.1M ammonium acetate (pH 4.6) containing 1.8% of acetonitrile and it was pumped at a flow-rate of 0.2 ml/min.

5-HT and 5-HIAA were measured by HPLC with electrochemical detection. Potential of the working electrode was 0.6 V. The mobile phase consisted of 0.1 M triethylamine, 0.1 M phosphoric acid, 0.3 mM EDTA, 8.2 mM heptane-1-sulfonic acid sodium salt, containing 2% of acetonitrile, and was pumped at a flow-rate of 0.25 ml/min; 2μl of the supernatant was injected into HPLC system for analysis. The intra-assay variation for TRP, 5-HT and 5-HIAA were 4–6%, while the inter-assay variation were 5–10%, respectively.

Serum concentration of 5-HT, 5-HIAA, and TRP was expressed in μM/L, nM/L, and μM/L, respectively. Additionally, 5-HIAA/5-HT ratio, as well as 5-HT/TRP ratio were calculated, as the indicators of the serotonin turnover.

### Serum biochemistry

The serum creatinine and urea concentrations were measured with commercial kit–CORMAY (Lublin, Poland); assays were performed using biochemical analyzer Minidray BS-120 (USA). The calcium and inorganic phosphorus were determined by Calcium arsenazo and Phosphorus kits from BioMaxima (Lublin, Poland). Parathyroid hormone (PTH) was analyzed using ELISA kit (Rat Intact PTH Elisa kit, Immunotopic, San Clemente, CA), whereas 1,25 dihydroxyvitamin D levels were determined using Rat (DHVD3) ELISA kit from Shanghai Sunred Biological Technology Co., Ltd, China. Serum markers of bone turnover: alkaline phosphatase (ALP) activity as bone formation marker and osteoclast-derived tartrate-resistant acid phosphatase form 5b (TRACP 5b) activity as bone resorption marker were measured using colorimetric commercial kits. ALP and TRACP 5b (RatTRAP™) assays were purchased from Biomaxima (Poland) and Immunodiagnostic Systems Ltd (Germany), respectively. Intra- and inter-assay coefficients of variation (CV) ranged from 2%–10%, respectively.

### Statistical analysis

Shapiro-Wilk’s test of normality was used for data distribution analysis. The normally distributed data were expressed as mean ± SD. The non-Gaussian data were presented as median (full range). The logarithmic transformation was applied to the not normally distributed data to ensure a normal distribution. The data were analyzed using a two-way analysis of variance (ANOVA). The two independent factors were: group (CKD or control) and age (1 or 3 months). If the ANOVA results showed significant differences (*p* < 0.05), post hoc Bonferroni test was used to test the level of significance between individual groups. The correlations between study variables were calculated by Spearman’s rank correlation analysis. A two-tailed p-value <0.05 was considered statistically significant. Computations were performed using Statistica ver.10 computer software (StatSoft, Tulsa, OK, USA). Graphic design presentation of results was performed using GraphPad Prism 6.0 software (USA).

## Results

### Serum biochemical parameters and weight gain

**Fig 2:**
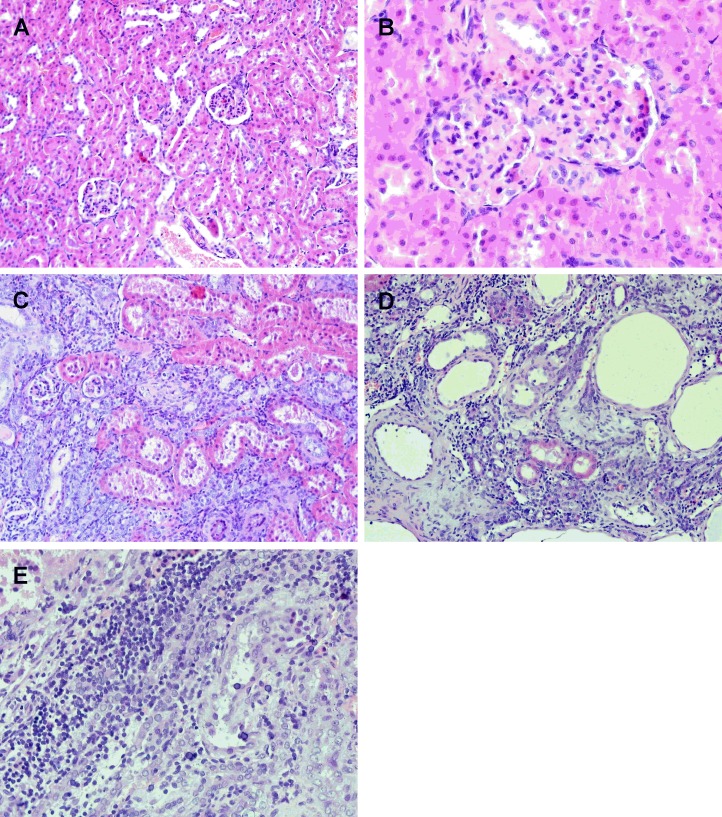
The histological changes in the kidney tissue 4 weeks after subtotal nephrectomy. (A) The intact kidney from sham-operated rat; (B-E) the renal stump of subtotal nephrectomized rat 4 weeks after CKD induction; (B) moderate glomerulosclerosis (C), strong tubular atrophy (D), focal interstitial fibrosis (E) focal interstitial lymphocytic infiltration (HE staining, magnification x200, x400).

Table 1 shows the general characteristics of studied groups. The 5/6 nephrectomy resulted in significant increase in creatinine and blood urea nitrogen (BUN) concentrations in CKD rats compared to healthy controls. There was no significant interaction of age and CKD on these parameters, but there were significant main effects of age or CKD on causing higher creatinine and BUN levels. Functional markers of CKD were associated with morphological changes in the remnant kidney. Fig 2 documents that 4 weeks after induction of CKD the moderate glomerulosclerosis, moderate to severe tubular atrophy, significant interstitial fibrosis, and focal interstitial lymphocytic infiltration were present in the remnant kidney. The average final body weight in the CKD-3 group was significantly lower than those in the CON-3 group. Two-way ANOVA analysis showed that both age and CKD had significant effects on the changes in body weight. Starting from the first month after 5/6 nephrectomy, the average of weight gains were significantly lower in CKD compared to appropriate controls. Two-way ANOVA revealed that the changes in the weight gain were due to an action of age and CKD as well as a result of an interaction between them. PTH levels were increased in the third month of CKD compared to the first month after subtotal nephrectomy. Moreover, the slight increase in PTH levels was noted in CKD-3 compared to appropriate controls. Two-way ANOVA analysis indicated that both age and CKD significantly altered serum PTH in these rats. Calcium concentrations were slightly decreased in CKD-3 rats compared to their counterparts, whereas the similar serum concentration of phosphorus was observed in all studied groups. 1,25-dihydrixyvitamin D levels were slightly decreased in CKD rats compared to appropriate controls. However, these changes were not statistically significant. The characteristics of CKD group were consistent with a state of mild CKD based on previously established criteria, i.e. increased serum creatinine and PTH concentrations, decreased calcium, and slightly but not statistical significantly decreased 1,25-dihydroxyvitamin D levels [59].

**Table 1:** Body weight and biochemical parameters in controls and chronic kidney disease (CKD) rats after one and three months of disease progression.

	CON, 1 month	CKD, 1 month	CON, 3 months	CKD, 3 months	Two-way ANOVA		
					age	CKD	interaction
Final body weight, g	278.43±12.51	254.00±14.24	384.00±32.21^^^	342.58±16.94**^,^^###^	p<0.0001	p<0.0001	NS
Weight gain, g	159.14±17.52	143.50±11.04*	274.30±25.05	234.33±20.00**^,^^###^	p<0.0001	p<0.01	p<0.05
Creatinine, μmol/l	27.40±2.65	38.90±6.19**	34.45±3.54	53.04±4.42***^,^^###^	p<0.0001	p<0.0001	NS
Food intake, g/day	23.70±2.60	22.86±4.78	29.32±4.58	20.41±2.31*	NS	p<0.05	NS
BUN, mmol/l	8.39±1.17	13.57±2.41**	6.66±0.43	11.89±1.68***	p<0.05	p<0.0001	NS
iP, mmol/l	2.21±1.51	2.29±1.81	2.09±0.83	2.14±1.02	NS	NS	NS
Ca, mmol/l	2.82±0.89	2.62±0.75	3.26±0.81	1.98±0.70*	NS	p<0.05	NS
PTH, pg/ml	256 (84–612)	300 (130–525)	320 (185–590)	490 (308–1338)*^,^^#^	p<0.01	p<0.05	NS
1,25 (OH)_2_ D, nmol/l	5.17±1.24	4.62±1.12	3.89±0.86	3.39±0.99	NS	NS	NS

BUN = blood urea nitrogen; iP = inorganic phosphorus; Ca = calcium; PTH = parathyroid hormone; 1,25 (OH)_2_D = 1,25 dihydroxyvitamin D; final body weight = weight at the time of sacrifice. Data are mean±SD or median (full range); n = 11 in each group.

*p<0.05

**p<0.01

***p<0.001, controls versus appropriate CKD group

^^^p<0.001 controls 1 month versus controls 3 months

^#^p<0.05

^###^p<0.001 CKD 1 month versus CKD 3 months

### Serotonin and its metabolite measurement

**Fig 3:**
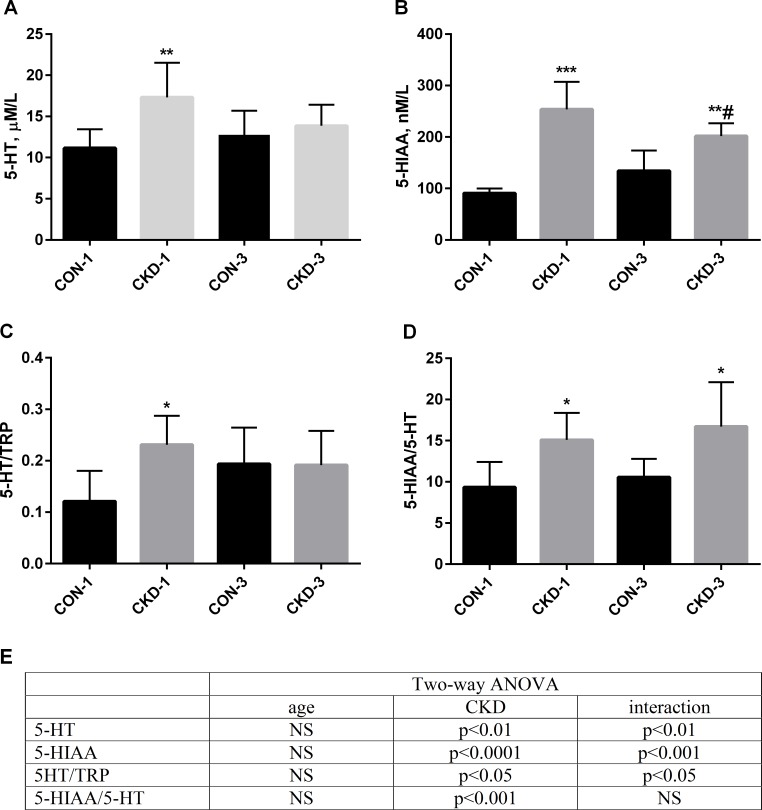
**The changes in 5-HT (panel A), 5-HIAA (panel B) levels, the ratio of 5-HT/TRP (panel C), the ratio of 5-HIAA/5-HT (panel D) and the results of two-way ANOVA with Bonferroni post hoc test (panel E) in controls and appropriate chronic kidney disease (CKD) rats.** Data are mean ±SD, n = 11 in each group. Abbreviations: CKD = Chronic Kidney Disease, CON = Controls, 5-HT = serotonin, 5-HIAA = 5-hydroxyindoleacetic acid, TRP = tryptophan; *p<0.05, **p<0.01, *** p<0.001 controls versus appropriate CKD group; #p<0.05 CKD-1 versus CKD-3.

There were no significant differences in serum TRP levels between CKD and control animals (CON-1: 77.9±9.6 μM/L, CKD-1: 75.7±12.1 μM/L, CON-3; 70.4±22.1 μM/L, CKD-3: 69.7±9.6 μM/L). As shown in Fig 3, subtotal nephrectomy resulted in increased levels of 5-HT and 5-HIAA in rat’s serum in comparison to control groups (panels A, B), which was mostly marked in rats sacrificed one month after surgery (CKD-1). Furthermore, we also observed higher 5-HT/TRP and 5HIAA/5-HT ratios in CKD animals, which indicate an intensified peripheral serotonin turnover (panels C, D). The concentrations of 5-HIAA in 24-hours urine collection were significantly higher in subtotal nephrectomized rats compared to their healthy counterparts (CKD-1: 450.22±125.83 nM/24 hours versus CON-1: 35.45± 13.22 nM/24 hours, p<0.0001 and CKD-3: 395.05±198.19 nM/24 hours versus CON-3: 47.41±16.91 nM/24 hours, p = 0.0008; respectively). Two-way ANOVA with Bonferroni post hoc test showed that subtotal nephrectomy alone and the interaction between nephrectomy and age of animals had a significant effect on increasing of these parameters (panel E).

#### Bone histological examination

**Fig 4:**
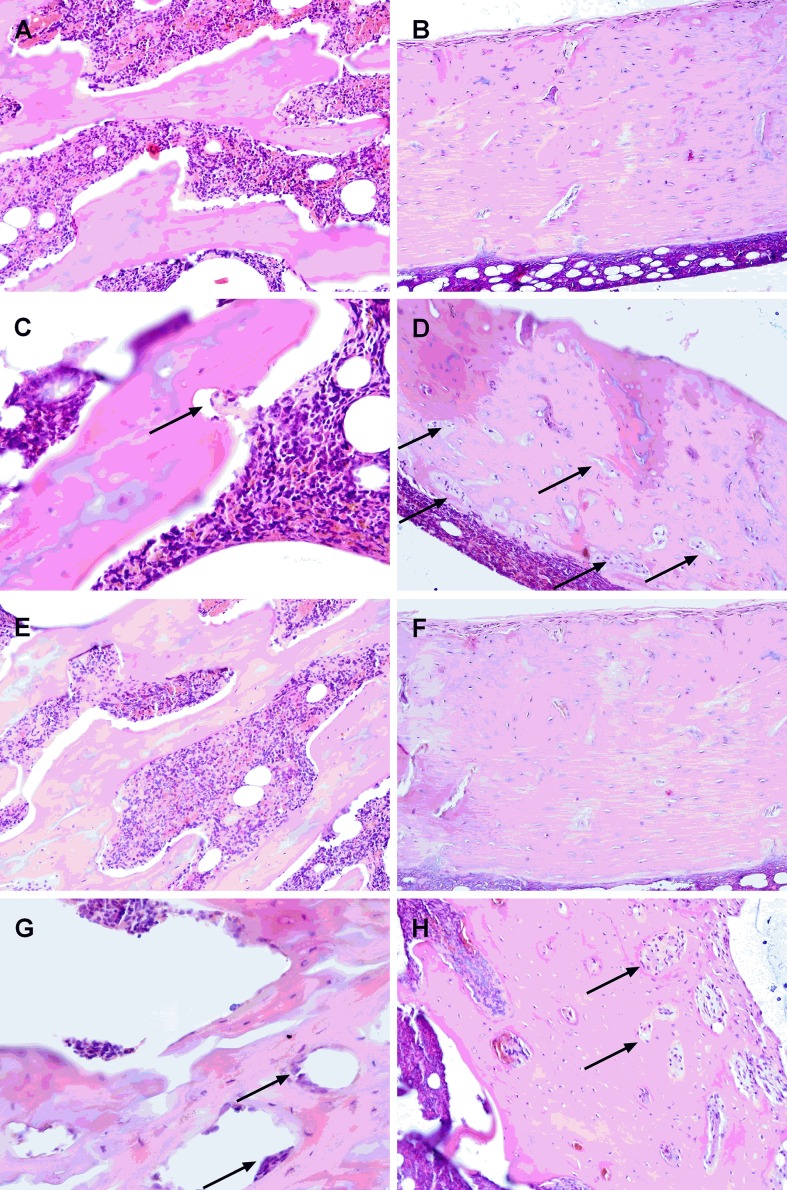
Representative images of the proximal tibia of controls and CKD rats after 1 and 3 months of CKD indication. (A) Spongy bone and (B) compact bone in tibia of the CON-1 rat; (C) Spongy bone of CKD-1 rat. Visible an increase in osteoclast surface and an increase in eroded surface of trabecular bone (arrow); (D) The increase in osteoclasts surface (arrows) in compact bone of CKD-1 rat; (E) Spongy bone and (F) compact bone in tibia of CON-3 rat; (G) The significant increase in the osteoclast surface (arrow) and the erosion of trabecular bone in CKD-3 rat; (H) areas of osteoclastic activity in compact bone (arrows) in CKD-3 rat (HE staining, magnification x200, x400).

The development of CKD went along with an increase of osteoclasts and osteoclastic erosion surface both one and 3 months after subtotal nephrectomy compared to shams. In the bone sections of rats one month after CKD induction about a 6-fold increase in the number of osteoclasts (1.29±0.12/mm^2^ in CKD-1 versus 0.22±0.08/mm^2^ in K-1, p<0.0001) and increased osteoclastic surface erosion have been observed (Fig 4), whereas CKD-3 animals presented a marked, 8-9- fold increase in the number of osteoclasts (1.86±0.14/mm^2^ in CKD-3 versus 0.21±0.07/mm^2^ in K-3, p<0.0001) and significantly increased erosion surface compared to controls. These changes were noted both in spongy and compact bone and the damage of trabecular bone was particularly seen (Fig 4).

#### The results of the three-point bending test and the bending test of the femoral neck

As shown in Table 2, the femoral length was independently affected by age both in CKD and in controls. However, age and CKD affected the biomechanical properties of the femur substantially. The stiffness in two studied bone sites was higher in the rats with the three-month of CKD development than in their counterparts. Two-way ANOVA with Bonferroni post hoc test revealed that the changes in the stiffness of femoral diaphysis were due to an action of age and CKD as well as a result of an interaction between them. In the case of the stiffness of the femoral neck, age and the interaction between age and CKD was significant (p< 0.05), but the effect of CKD alone was not. F_u_ and dl (F_u_) in two studied bone sites remained unchanged in CKD compared to appropriate controls. However, the significant decrease in dl (F_u_) was observed in older compared to younger CKD as well as healthy animals. Two-way ANOVA with Bonferroni post hoc test revealed that the changes in this parameter were due to an effect of age and CKD. F_y_ was significantly decreased at the level of femoral diaphysis and femoral neck starting from the first month after 5/6 nephrectomy. At the diaphysis, F_y_ increased together with the CKD development, reaching values similar to controls in the 3rd month of CKD. In contrast, F_y_ values at the femoral neck remained reduced during CKD development. Two-way ANOVA with Bonferroni post hoc test revealed that the changes in this parameter were related to CKD. The values of dl (F_y_) at the level of diaphysis were similar in uremic and healthy animals, whereas dl (F_y_) at the level of the femoral neck was lower in CKD-3 than in a K-3 group. As shown by the analysis of two-way ANOVA, the values of dl (F_y_) significantly decreased with animals’ age in both CKD and control rats (p<0.001) at the level of the femoral neck. At the diaphysis, the values of W were higher in CKD than in controls in the 3rd month of CKD. At the femoral neck, bones from CKD-1 and CKD-3 animals absorbed less energy before fracture compared to appropriate controls, and this property diminished with animals’ age. Two-way ANOVA with Bonferroni post hoc test showed that the decrease in W at the femoral neck was due to an effect of age and CKD.

**Table 2:** Femoral length and biomechanical properties of the femur of control and chronic kidney disease (CKD) rats evaluated by the three-point bending test (A) and the bending test of the femoral neck (B).

	CON, 1 month	CKD, 1 month	CON, 3 months	CKD, 3 months	Two-way ANOVA		
					age	CKD	interaction
Femoral length, mm	29.07±0.40	29.05±0.97	33.59±1.23^^	34.03±1.72^###^	p<0.0001	NS	NS
Stiffness, N/mm (**A**)	191.69±26.38	166.74±31.31	265.91±59.69	310.93±50.78*^,^^###^	p<0.0001	p<0.05	p<0.05
Stiffness, N/mm (**B**)	133.58±45.17	105.05±10.50	149.98±53.19	184.50±43.72*^,^^###^	p<0.001	NS	p<0.05
F_u_, N (**A**)	108.79±10.43	96.94±10.63	161.67±18.88^^	151.35±30.87^###^	p<0.0001	NS	NS
F_u_, N (**B**)	157.17±20.85	133.77±29.92	166.52±30.65	164.91±44.48^#^	p<0.05	NS	NS
dl (F_u_), mm (**A**)	0.64±0.14	0.80±0.08	0.59±0.11	0.62±0.18^#^	p<0.05	p<0.05	NS
dl (F_u_), mm (**B**)	1.46±0.35	1.18±0.19	1.20±0.48	0.86±0.19^#^	p<0.01	p<0.01	NS
F_y_, N (**A**)	42.95±9.17	25.26±8.82**	98.87±18.39^	77.92±23.18^##^	p<0.001	p<0.001	NS
F_y_, N (**B**)	125.02±12.46	93.80±6.04**	121.75±6.79	90.10±11.56**	NS	p<0.001	NS
dl (F_y_), mm (**A**)	0.22±0.02	0.24±0.06	0.27±0.09	0.32±0.06^#^	p<0.05	NS	NS
dl (F_y_), mm (**B**)	1.04±0.39	0.91±0.12	0.87±0.26^	0.52±0.15*^,^^##^	p<0.001	p<0.01	NS
W, mJ (**A**)	35.00±9.26	48.18±8.74	44.29±11.33	59.09±13.00*	p<0.01	p<0.001	NS
W, mJ (**B**)	55.46±15.98	37.27±6.51**	41.71±10.82^	18.73±5.78**^,^^###^	p<0.0001	p<0.0001	NS

Abbreviations: F_y_ = yield load; dl (F_y_) = displacement at the yield load; F_u_ = ultimate load; dl (F_u_) = displacement at the ultimate load; W = work to fracture. Data are mean±SD, n = 11 in each group.

*p<0.05

**p<0.01

***p<0.001 controls versus appropriate CKD group

^p<0.05

^^p<0.01

^^^p<0.001 controls 1 month versus controls 3 months

^#^p<0.05

^##^p<0.01

^###^p<0.001, CKD 1 month versus CKD 3 months

At the level of the femoral diaphysis, the stiffness was positively correlated with F_u_ and F_y_ (R = 0.542, p<0.01 and R = 0.748, p<0.00; respectively), whereas it was inversely related to dl (F_u_) and W (R = -0.830, p<0.0001 and R = -0.521, p<0.05; respectively). The positive correlation was between F_u_ and F_y_ (R = 0.688, p<0.001), whereas the tendency to inverse correlation was between F_u_ and dl (F_u_), R = -0.337, p = 0.074. Moreover, the inverse correlation was between F_y_ and W (R = -0.490, p<0.05).

At the level of the femoral neck, the stiffness was inversely related to dl (F_y_), dl (F_u_) and W (R = -0.705, p<0.001, R = -0.618, p<0.01 and R = -0.605, p<0.01; respectively).

### Serum bone turnover markers

**Fig 5:**
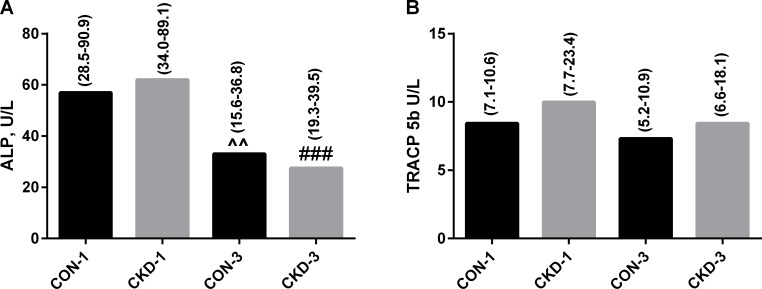
The serum ALP and TRAC 5b activities in controls and appropriate chronic kidney disease (CKD) rats. Data are presented as median (full range), n = 11 in each group. Abbreviations: CKD = Chronic Kidney Disease, CON = Controls, ALP = alkaline phosphatase, TRACP 5b = tartare-resistant acid phosphatase form 5b; ^^ p<0.01 controls 1 month versus controls 3 months; ### p<0.001 CKD-1 versus CKD-3.

As has been shown in Fig 5, there were no differences in the serum ALP activity between CKD and controls, although the age-dependent decrease in this bone formation parameter was observed both in uremic and control groups. TRACP 5b activity showed a tendency to increase in CKD-1 animals compared to appropriate controls, and this parameter of bone resorption was independent from age. Two-way ANOVA with Bonferroni post hoc test revealed that age but not CKD had a significant effect on the changes in the ALP activity (p<0.0001). Moreover, ALP was not associated with TRACP 5b activity both in CKD and control rats.

### Relationships between 5-HT, its metabolite and biomechanical parameters of femur

We distinguish a number of statistically significant correlations between serotonin, its metabolite and biomechanical parameters of the left femur of CKD rats, obtained from both biomechanical tests (Table 3). At two studied bone sites, both 5-HT and 5-HIAA were inversely correlated with stiffness. Moreover, 5-HT and its metabolite correlated positively with dl (F_y_) and dl (F_u_). At the level of the femoral diaphysis, both 5-HT and 5-HIAA were inversely related to F_y_, whereas TRP and 5-HIAA levels were positively correlated with W. At the level of the femoral neck, both 5-HT and 5-HIAA were positively related to W, and 5-HIAA was positively correlated with F_y._

**Table 3:** The correlations between TRP, 5-HT, 5-HIAA and biomechanical properties of the femur of CKD rats: A–three-point bending test (n = 22), B–bending test of the femoral neck (n = 22).

	A			B		
	TRP	5-HT	5-HIAA	TRP	5-HT	5-HIAA
Stiffness	R = -0.238 NS	R = -0.518 p = 0.020	R = -0.575 p = 0.007	R = -0.263 NS	R = -0.421 p = 0.043	R = -0.500 p = 0.023
F_u_	R = -0.009 NS	R = -0.307 NS	R = -0.385 NS	R = -0.376 NS	R = -0.153 NS	R = -0.277 NS
dl (F_u_)	R = 0.428 p = 0.048	R = 0.405 p = 0.023	R = 0.595 p = 0.005	R = 0.228 NS	R = 0.475 p = 0.034	R = 0.528 p = 0.019
W	R = 0.428 p = 0.047	R = 0.195 NS	R = 0.453 p = 0.030	R = 0.016 NS	R = 0.478 p = 0.033	R = 0.565 p = 0.009
F_y_	R = -0.303 NS	R = -0.463 p = 0.036	R = -0.592 p = 0.005	R = -0.039 NS	R = 0.157 NS	R = 0.462 p = 0.040
dl (F_y_)	R = 0.336 NS	R = 0.538 p = 0.019	R = 0.583 p = 0.004	R = 0.415 p = 0.047	R = 0.435 p = 0.037	R = 0.515 p = 0.021

Abbreviations: TRP = tryptophan; 5-HT = serotonin; 5-HIAA = 5-hydroxyindoleacetic acid; CKD = chronic kidney disease; F_y_ = yield load; dl (F_y_) = displacement (deformation) at the yield point; F_u_ = ultimate load; dl (F_u_) = displacement (deformation) at ultimate load; W = work to fracture; NS = not significant.

In the controls, there was an inverse relationship only between F_u_, F_y_ and 5-HIAA levels in the bending test of the femoral neck (R = -0.665, p = 0.013; and R = -0.852, p<0.001, respectively), while 5-HT and its metabolite did not correlate with any of the biomechanical parameters at the level of the femoral diaphysis.

### Relationships between 5-HT, its metabolite and bone turnover markers

**Fig 6:**
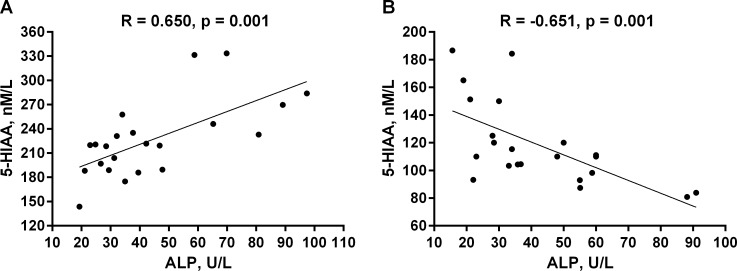
**The correlation between ALP and 5-HIAA serum levels in rats with CKD (panel A) and controls (panel B).** Abbreviations: ALP = alkaline phosphatase, 5-HIAA = 5-hydroxyindoleacetic acid.

In CKD animals, 5-HT tended to be positively correlated with serum ALP activity (R = 0.419, p = 0.058). The positive correlation between 5-HIAA and ALP activity was shown in Fig 6A. Serum creatinine concentrations were inversely correlated with 5-HT (R = -0.520, p = 0.01). We also distinguish the positive association between 5-HT and its metabolite 5-HIAA (R = 0.687, p<0.001).

In contrast to CKD animals, the inverse relationship was between 5-HIAA and ALP activity in controls (Fig 6B). The positive relationships were noticed between 5-HT, 5-HIAA levels and creatinine concentrations (R = 0.607, p = 0.047; and R = 0.804, p = 0.003; respectively).

There were no correlations between TRACP 5b activity and the serotonergic pathway metabolites in CKD as well as in control animals.

### Relationships between bone turnover markers and bone biomechanics

The correlations between bone formation/resorption markers and the results of the three-point bending test and the bending test of the femoral neck in CKD are presented in Table 4. ALP activity was inversely correlated with cortical stiffness and strength, but it was positively related to the displacements. TRACP 5b activity was inversely correlated with F_u_ and W values. Regarding ALP activity, the similar results were obtained in the bending test of the femoral neck, in which ALP activity was furthermore positively correlated with the capability to energy absorption (W). There was no relationship between TRACP 5b activity and the parameters of the bending test of the femoral neck.

**Table 4:** The correlations between serum alkaline phosphatase (ALP) and tartrate-resistant acid phosphatase form 5b (TRACP 5b) activities and biomechanical parameters of the femur of CKD rats: A–three-point bending test (n = 22), B–bending test of the femoral neck (n = 22).

	A		B	
	ALP	TRACP 5b	ALP	TRACP 5b
Stiffness	R = -0.738 p<0.001	R = -0.225 NS	R = -0.753 p<0.001	R = -0.318 NS
F_u_	R = -0.542 p = 0.007	R = -0.468 p = 0.033	R = -0.432 p = 0.039	R = -0.205 NS
dl (F_u_)	R = 0.687 p<0.001	R = -0.052 NS	R = 0.588 p = 0.004	R = 0.203 NS
W	R = -0.175 NS	R = -0.472 p = 0.031	R = 0.762 p<0.001	R = 0.359 NS
F_y_	R = -0.677 p<0.001	R = -0.253 NS	R = 0.114 NS	R = 0.258 NS
dl (F_y_)	R = 0.720 p<0.001	R = 0.089 NS	R = 0.725 p<0.001	R = 0.371 NS

Abbreviations: CKD = chronic kidney disease; F_y_ = yield load; dl (F_y_) = displacement (deformation) at the yield point; F_u_ = ultimate load; dl (F_u_) = displacement (deformation) at ultimate load; W = work to fracture; NS = not significant.

In controls, the inverse relationship was only observed between ALP activity and cortical stiffness (R = -0.709, p = 0.015). However, the inverse correlation was between TRACP 5b activity and cortical stiffness and F_u_ (R = -0.629 and R = -0.657, both p<0.05; respectively). Moreover, both ALP and TRACP 5b activities were not related to parameters of the bending test of the femoral neck.

## Discussion

The animal and human studies shown that CKD leads to osteodystrophy and/or osteoporosis [26–35, 60]. There are also data obtained previously by us [36–38] and by others [26, 40–45] indicated the disturbances of the peripheral serotonergic system in patients with CKD and experimental CKD models [39, 45–46]. In the present study, we demonstrate for the first time that: 1) the mild degree of CKD can exert the distinct effect on biomechanical properties of cortical and mixed cortico-trabecular bone; 2) impaired renal function affects the peripheral serotonin metabolism, which in turn may influence the strength and metabolism of long bones in these rats.

The results of our study showed that renal insufficiency substantially affected peripheral serotonin metabolism in rats with CKD, particularly in the early period after nephrectomy. At this time, serum 5-HT and its metabolite– 5-HIAA concentrations, as well as urinary 5-HIAA excretion, rose significantly in CKD group compared to controls. These data are consistent with the previous clinical and experimental observations [40–41, 44, 45]. The elevation in peripheral serotonin levels may be a result of increased synthesis or reduced its catabolism. As has been shown in Fig 3, 5-HT/TRP ratio, which reflects Tph-1 activity–the rate-limiting enzyme in the 5-HT biosynthetic pathway, was significantly higher in CKD-1 animals compared to controls. The bulk of peripheral 5-HT is metabolized by endothelial cells to 5-HIAA [44]. The 5-HIAA/5-HT ratios, reflecting the rate of 5-HT metabolism into 5-HIAA, were significantly higher during CKD development. Above data indicate intensified peripheral serotonin turnover in uremia, and are in agreement with the previous observation of Ksiazek et al. [45]. As has been shown in Fig 3, CKD alone and its interaction with age appear to be responsible for this effect. In the case of decreased glomerular filtration rate, reflecting by increased serum BUN and creatinine concentrations, the elimination of 5-HT and 5-HIAA is reduced, resulting in increased their levels in serum. These findings are consistent with the results of a study performed on uremic patients [44].

The bone quality can be quantitatively estimated in biomechanical tests based on the bone strength (F_y_, F_u_), stiffness and ability to absorb energy (W) [57–58]. In the present study, these properties are determined by two different bone type: the cortical bone of the femoral diaphysis using three-point bending test and the mixed cortico-trabecular structure of the proximal femur by the bending test of the femoral neck. The very young, rapidly growing rats were used, in which the interference of the general growth process makes the exact determination of CKD-dependent skeletal effects difficult to determine. For this reason, two-way ANOVA was employed to isolate between-group differences in the studied parameters.

The observed changes in the biomechanical properties of the femur were mostly due to an influence both age and CKD (Table 2). The results of the biomechanical tests showed the distinct effect of CKD on bone strength, depending on the bone site. During three-month development of CKD, the pure cortical bone (femoral diaphysis) became more rigid than in controls, however, the other biomechanical parameters determining the whole-bone strength: F_y_, F_u_ and W were similar between groups. So far, there are few studies, in which authors assessed the quality of the bone in rats with subtotal nephrectomy using three-point bending test [47–48]. Jokihaara et al. [48] observed no differences between CKD animals and controls in F_u_, stiffness and W of the femoral shaft, despite the decrease in bone mineral density (BMD). Iwamoto et al. [47] also reported that W and F_u_ did not differ significantly among CKD animals and controls. The results of the present study are in agreement with previous observations [47–48] and indicate the preserved cortical bone strength in rapidly growing rats with a mild degree of CKD. These data suggest that an early adaptive response related to growth in the young rats could provide protection from the deleterious effects of CKD on cortical bone strength. A similar effect of intense growth on bone strength preservation was previously described by Lambert et al. [56] in young rats with dietary restriction.

The results of the bending test of the femoral neck revealed that the stiffness and F_u_ of the mixed cortico-trabecular bone behaved like those in cortical bone. However, in contrast to the cortical bone, the energy absorption capacity (W) reflecting bone toughness and yield load (F_y_) in this bone site was significantly lower in CKD compared to control animals. These features are crucial in bone biomechanics because a tough bone is more resistant to fracture, even though it may be less resistant to yield load. Lower F_y_, reduced toughness, and higher bending stiffness suggest that femoral neck in uremia is more brittle and less tough compared to healthy bone, and may be more susceptible to fracture under trauma than femoral diaphysis. These data are in agreement with the results of the histological bone examination, which showed more severe damage of trabecular than cortical bone in rats with CKD. While Jokihaara et al. [48] described the lack of the changes in the results of the compression test of the femoral neck between CKD and control rats. These discrepancies may result from differences in age and strain of animals used in both experiments.

The distinct effect of CKD on biomechanical properties of virtually pure cortical (femoral diaphysis) and mixed cortico-trabecular bone (femoral neck) noticed in this study can be caused by many reasons. Cortical bone is dense and surrounds the marrow space, whereas trabecular bone is 20 to 30% less stiff than cortical, and is localized mainly in the epiphysis of long bones and vertebras [31]. They differ not only in the tissue micro-architecture, but also in their cellular contents, metabolic rates, and bone marrow constituents. Moreover, the bone metabolic activity is higher in trabecular than in cortical bone, making trabecular type more suitable to evaluate early biochemical changes of bone metabolism [61]. All these differences between trabecular and cortical bone tissue can probably be a cause of distinct results of the biomechanical tests performed on these two bone types in rats with CKD.

As has been shown in Table 3, the elevated peripheral serotonin metabolism is substantially associated with biomechanical properties of the cortical and cortico-trabecular bone of young rats with CKD. Both 5-HT and 5-HIAA were inversely associated with stiffness, whereas they were positively associated with displacements and toughness in both types of bone. However, the opposite effect was observed in the case of F_y_, which was inversely associated with 5-HT and its metabolite in cortical bone, whereas it was positively correlated with 5-HIAA levels in cortico-trabecular bone. These findings suggest the relationship between 5-HT metabolism and bone elasticity and stiffness, making the bone less brittle. In the conditions of the present study, the impact of the serotonergic system on the cortico-trabecular part of the femur seems to be beneficial. However, the strength of the cortical bone could be potentially reduced by the unfavorable effect of 5-HT and its metabolite on F_y_, making the bone more susceptible to microcracking.

As has been presented in Fig 5, bone formation biomarker was similar in CKD and control animals, whereas bone resorption biomarker showed a tendency to increase, which was particularly evident in the early period after nephrectomy. For evaluation of bone metabolism, we chose biomarkers (ALP, TRACP 5b) which removal from the circulation is independent of renal function [62]; this allows for precise assessment of bone metabolism in the conditions of renal failure. In the present study, we observed the opposite effect of the peripheral serotonergic system on bone formation process in CKD and healthy rats (Fig 6). Under physiological conditions, 5-HIAA levels, reflecting the total amount of circulating 5-HT, were inversely associated with ALP activity. This finding is in agreement with results Yadav et al. [10–12], who demonstrated that peripheral 5-HT is a powerful inhibitor of bone formation. However, increased 5-HIAA values were positively associated with bone formation in CKD animals. Recently, Dai et al. [63] showed that serotonin can exert a dual effect on bone formation: at low concentrations 5-HT can impair, whereas at higher concentrations it showed a trend to promote bone formation. The presented results seem to be consistent with this observation. However, indirect effects of serotonin and other neurotransmitters or hormones affecting bone metabolism can’t be ruled out. Moreover, we used intensive growing rats in this study, and it is possible that serotonin can play a special role in bone development in such young organism, characterized by a rapid bone accrual. In contrast to a significant role of peripheral serotonin in the modulation of bone formation, bone resorption process was not associated with 5-HT and its metabolite in this study, and this is in agreement with the previous observations of Yadav et al. [10–12].

Bone strength is determined by its overall structure and material integrity, which are dependent on bone cells metabolism [64]. As has been shown in Table 4, the serotonin-dependent bone formation was associated with a decrease in stiffness and strength, whereas this process was related to the higher elasticity at the level of cortical bone. However, serotonin-independent bone resorption can also affect F_u_ and W at this bone place, making the net impact of 5-HT on cortical bone difficult to determine. In contrast, serotonin-dependent bone formation inversely affected cortico-trabecular stiffness and F_u_ but improved toughness and elasticity. These data suggest that serotonin-dependent bone formation have a significant impact on bone biomechanical properties in rats with CKD, and they complement the results presented in Table 3.

Still now, there are no data about the effect of peripheral 5-HT and 5-HIAA on bone metabolism and strength in CKD. The results of the only one clinical study revealed that peripheral 5-HT was positively associated with bone formation and resorption biomarkers in hemodialysis patients [26]. In an experimental model, subcutaneously administration of 5-HT into healthy 2-month-old female Sprague-Dawley rats for 3 months resulted in the increase of stiffness and energy absorption before a three-point bending tests of bones [25]. In postmenopausal osteoporosis model, where a significant increase in the expression of circulating serotonin was observed, low-magnitude high-frequency loading improved the stiffness and strength of femoral bone by reducing of circulating 5-HT levels [15]. The above data suggest that the discrepancies concerning the impact of the peripheral serotonin on bone strength could depend on the different skeletal sites examined, age, species, and sex of studied organism as well as the pathomechanism of bone disease.

Our study has several limitations. First, we had no possibility to measure BMD in this experiment. However, the previous study [48] showed that BMD does not reflect the bone strength in 5/6 nephrectomy model. Secondly, the serum levels of bone formation and resorption biomarkers are probably not an exact reflection of their tissue levels. Thirdly, the causal relationship between peripheral 5-HT and bone strength cannot be established owing to the cross-sectional design of the study. Moreover, although experimental factors, such as age, race, sex, pathogenic mechanism, lack of medication and living environment are well controlled in animal models, their biological response may differ from that of human due to the differences in rat versus human cortical structure, and the lack of intracortical bone remodeling in rats [65].

## Conclusions

In contrast to the several studies demonstrated adverse effects of CKD on bone in rats [30–32, 60], the results of the present study indicate the distinct effect of mild CKD on biomechanical properties of the femur. An early adaptive response related to growth in the young rats can provide protection from the deleterious effects of CKD on cortical bone strength. However, the strength of mixed cortico-trabecular bone was impaired compared to healthy bone. Moreover, this study for the first time demonstrates the association between elevated peripheral serotonin and its metabolite, bone metabolism and biomechanical properties in growing rats with mild CKD. The distinct impact of the serotonergic system was observed, depending on the type of bone–the effect seems to be beneficial on the properties of the cortico-trabecular bone, whereas the strength of the pure cortical bone could be potentially reduced. The serotonin-dependent bone formation can have a significant influence on bone biomechanical properties in growing rats with CKD. The present study adds the new information to the relatively scarce clinical data about the effect of peripheral serotonin on bone metabolism, and may contribute to a better understanding of CKD-BMD pathophysiology. Although there are some limitations on the animal model of CKD, the present results seem to be applicable to children and adolescents with a mild degree of CKD.
